# Supplementary material for: Depressed Mood as a Significant Risk Factor for Gynecological Cancer Aggravation
Source: Int J Environ Res Public Health. 2023 Oct 2;20(19):6874. doi: 10.3390/ijerph20196874 (PMC10573065; doi:10.3390/ijerph20196874)
Supplement: Supplementary file 1 [file ijerph-20-06874-s001.zip › ijerph-2584421-supplementary.pdf]

Supplementary Materials

Table S1. Other characteristics and Cox proportional hazards of DFS in univariate analysis for each group not shown in Table 1.

	**Non-Depressed Mood (N = 129)**	**Depressed Mood (N = 88)**	***p*-value**	**Risk factors for DFS**		
				**HR**	**95% CI**	***p*-value**
Height (cm), M±SD	155.29±5.58	155.57±6.22	0.723	1.00	(0.95, 1.05)	0.979
Weight (kg), M±SD	59.09±12.21	56.43±10.61	0.100	0.99	(0.97, 1.01)	0.409
BMI (kg/m^2), M±SD	24.56±4.99	23.56±4.70	0.140	0.99	(0.94, 1.04)	0.694
Parity status, N (%)			0.260			
Parity = 0	31 (24.0%)	28 (31.8%)		1.00	Reference	
Parity = 1	19 (14.7%)	6 (6.8%)		1.14	(0.44, 2.98)	0.787
Parity = 2	51 (39.5%)	35 (39.8%)		1.19	(0.61, 2.32)	0.606
Parity > 3	28 (21.8%)	19 (21.6%)		1.29	(0.61, 2.71)	0.502
Drinking status, N (%)			0.397			
No	127 (98.4%)	85 (96.6%)		1.00	Reference	
Yes	2 (1.6%)	3 (3.4%)		0.05	(0.00, 67.95)	0.408
Smoking status, N (%)			0.567			
No	128 (99.2%)	86 (97.7%)		1.00	Reference	
Yes	1 (0.8%)	2 (2.3%)		0.05	(0.00, 43.19)	0.377
Number of underlying diseases, N (%)			0.930			
0	80 (62.0%)	51 (58.0%)		1.00	Reference	
1	36 (27.9%)	28 (31.8%)		1.16	(0.65, 2.04)	0.619
2	10 (7.8%)	7 (8.0%)		0.70	(0.25, 1.98)	0.500
≥ 3	3 (2.3%)	2 (2.2%)		0.88	(0.12, 6.43)	0.899

Note: Values are presented as mean±standard deviation or N (%). M±SD, mean±standard deviation.
